# Twelve Tips for teaching clinical and communication skills online

**DOI:** 10.15694/mep.2021.000027.1

**Published:** 2021-01-29

**Authors:** Helen Bintley, Graham Easton, Riya George, Devina Raval, Harvey Wells, Nisha Ehamparanathan, Harriet Le Voir, S. Elizabeth Wright, Dason Evans, Angela Rowlands, Ashia Shafi

**Affiliations:** 1 Queen Mary University of London

**Keywords:** Online, clinical skills, communication skills, pedagogy, technology

## Abstract

This article was migrated. The article was marked as recommended.

Teaching and learning online is a long-established pedagogical approach in medical education. However, the Covid-19 pandemic has escalated the use and development of online teaching and with it have come a number of benefits and challenges. In this article the authors consider these benefits and challenges in relation to the teaching and learning of clinical and communication skills, drawing on up-to-date evidence and their extensive experience of using online learning before and during the Covid-19 pandemic.

The authors have identified twelve tips to support others in constructing and developing online clinical and communication skills for medical students taking into account educational vision and curriculum, the educational multi-disciplinary team, feedback and evaluation, and what to do when things go wrong. This article provides a practical guide to teaching clinical and communication skills in a new learning environment, which is likely to be a much more prominent feature of medical education in the future.

## Introduction

The COVID-19 pandemic has not only had a significant impact on society as a whole but also on how education is delivered across the UK. Described by Sandars *et al*., (2019, p. 2) as “not one single entity but rather a growing collection of modalities and technologies” online learning has become a matter of necessity for medical education. Despite these possibilities, online learning poses challenges that accompany such a shift in educational approach. For instance, such a shift can isolate people and merge the boundaries between work and home, but also provide a flexible, individualised environment for learners ( Rose, 2020). Furthermore, it is important to consider the pedagogical impact of moving education online and to ensure that any new approaches to teaching and learning are underpinned by relevant evidence and scholarship.

As is the case with many practical disciplines, clinical and communication skills have been challenging to deliver online. Teaching and learning clinical and communication skills requires a significant amount of person-to-person contact and relies on a variety of skills that are difficult to reproduce in the virtual environment. Furthermore, students don’t always have access to appropriate technologies or teaching spaces when learning remotely and this can impact on their educational experience ( Matthew Seah, 2020). Since lockdown, examples have emerged of creative ways to teach these kinds of skills online, including ophthalmoscopy ( Co Shih *et al*., 2020) and undergraduate practical clinical skills ( Wallace, 2020). Common challenges that emerge from these examples include staff engagement with established technologies and the need for flexibility in approaches to teaching and assessment. This requires evolving approaches to unstable circumstances, particularly in a subject in which human contact is so vital for competency. This need for flexibility in the face of change mirrors the constantly adapting clinical setting and working lives of healthcare professionals, especially during the pandemic. This shift in approach could be seen as an opportunity to role model to students strategies for managing uncertainty in the clinical environment as well as mirroring likely post-COVID changes to the clinical environment such as the increased use of remote consulting.

It is also important to recognise that the shift to online learning in medical education is not new and did not emerge during Covid-19. Online elements of medical curriculum are long established and innovative learning packages such as virtual patients and MOOCs (Massive Open Online Courses) have been used to teach a number of subjects in undergraduate and postgraduate medicine ( Cenden and Lok, 2012; Ilgen, Sherbino, and Cook, 2013; Swinnerton *et al*., 2016). Even so, these interventions are not without their difficulties. For instance, the effective integration of MOOCs into curricula and online platforms is complex, as are issues surrounding student engagement ( Park and Choi, 2009; Pickering *et al*., 2017; De Jong *et al*., 2020), and the effect of virtual patients on learning outcomes is uncertain and under-researched ( Cook, Erwin, and Triola, 2010; Ilgen, Sherbino, and Cook, 2013; Kononowicz *et al*., 2019); this limits their use in a wider context. In the short term, therefore, the pandemic has *accelerated* rather than initiated shifts in some areas from predominantly face-to-face modes of learning to online learning. With this move comes a previously understated commitment from higher education providers to find solutions to the issues online learning presents, particularly for practical subjects such as clinical and communication skills. This commitment will likely require all those involved in curriculum development to innovate, drawing on (and in the process critiquing) learning theories such as connectivism ( Siemens, 2005; Scott Goldie, 2016) and blended learning for the creation of high quality online learning materials for medical students.

The following tips have been assembled by the members of one teaching team in a clinical and communication skills unit in a UK medical school. They draw on their extensive experience of using technology-enhanced learning to offer advice for use in and beyond the current situation.

## Tip 1: Make student learning your top priority

It is easy to be distracted by the draw of novel technologies and the needs of faculty in this new landscape; but the primary focus must be your students. Remote student learning requires flexibility in approach to suit the need of the students, with regular updates and modifications ( Childs *et al*., 2005). Incorporate flexibility into the design of courses so that students from different time zones or locations can participate in asynchronous online discussion groups ( Cook, 2007). It is also important to support students by contextualising their learning and directing them to prioritise and understand the relevance of clinical and communication skills learning ( Roberts, Newman, and Schwartzstein, 2012). Remote delivery of clinical and communication skills should take into consideration mediums that support students develop deeper approaches to learning ( Gormley *et al*., 2009).

For example, providing formative feedback can support students’ development and help them to reach their anticipated performance goals ( Ende, 1983). It is therefore important to incorporate opportunities for feedback during learning activities ( Perera *et al*., 2008). This can be during small group discussions during synchronous online learning activities or using asynchronous activities such as a discussion forum. Providing students with facilitated opportunities to discuss and reflect what they have understood enables them to learn about clinical skills online more effectively ( Gormley *et al*. 2009).

## Tip 2: Be clear about what you expect students to do

Much of the pedagogy behind teaching clinical and communication skills stems from social learning theories, which emphasise the importance of acquiring knowledge and skills through observation, practice and feedback and to scaffold learning (continually building upon existing knowledge through reflection and discussion). Optimising the use of online videos and simulations for instance can encourage individual reflection and group discussions as well demonstrate essential procedural and communication skills with peer and educator feedback ( Goh and Sandars, 2019). But students need explicit guidance on what is expected of them in this new learning landscape.

Establishing clear and accessible channels of navigation and communication are essential to guide learners and educators through the online learning journey ( Taha *et al.,* 2020). We recommend explicit communication about expectations, both what learners should expect during their course of study and the responsibilities of the learners themselves (e.g. how they should participate in online forums). Additionally, mixing a blend of asynchronous and synchronous activities, and opting for co-creation between students and teachers is ideal to maximise student engagement. Using assessment tools as learning tools, rather than a ‘jump through the hoops’ activity can also be employed online. We recommend a blend of summative (for example, virtual role-plays and reflective essays) and formative (for example, describing procedural skills or practicing role plays between peers) assessments, constructively aligned to the content of the teaching material to support students’ learning.

## Tip 3: Innovate using institution-supported eLearning technologies

When creating online learning for students, the vast range of complex technologies and online platforms can seem like a huge barrier to innovation. However, in our experience it is well worth making the effort to overcome this barrier. Programs such as H5P ( Moodle, 2020) provide succinct navigation tools, making large and complex modules easy to view and interact with. Technology like H5P can also enable patients’ stories to be voiced in innovative ways, providing a space for transforming students’ understanding of the patient experience ( Christiansen, 2011; Matthews, 2014).

We recommend exploring the technological options available to you and to work with your institution’s elearning unit, who should be able to guide you in setting up and developing resources using established technologies. This is the approach we took with H5P in our online learning development during the pandemic. We identified a need, which was primarily to make a large amount of online content easier to navigate. We then identified that H5P, which was supported by our institution, could be used to address this need. We then identified one member of the team to be trained by the elearning unit to implement H5P into our learning resources to aid navigation through the module and develop and deliver training on H5P to other members of the team. This was all achieved in 2 months and was a very valuable experience for both students and staff.

## Tip 4: Make sure that clinical and communication skills content are appropriately integrated

By using the *complementary* skill sets of team members it is possible to deliver more integrated online teaching sessions for students. We found that clinical and communication skills in this way can create an opportunity for the integration of pathology, physiology, anatomy, examination alongside communication, facets of informed consent, role of chaperones, ethics and cultural considerations for intimate examinations. Clinical and communication skills can then be applied to other examinations and procedures, promoting good medical care ( Nobel *et al*., 2018) despite the limitations that online learning can pose.

From our experience of using this integrated approach in our online learning we advise that timetabling is clear, with staff members given plenty of time to prepare for any integrated session they are involved with. Alongside this, for each session the objectives and expectations of each staff member need to be made clear at the outset. Any concerns of non-clinical academic staff contributing to traditionally clinically orientated sessions need to be acknowledged and prioritized with the limitations of remote practice.

## Tip 5: Give students the opportunity to practice clinical and communication skills remotely

Teaching clinical and communication skills is vital for developing competence in students, but it is particularly difficult to deliver remotely. The COVID pandemic has prompted medical educationalists to consider this problem urgently, with some creative suggestions including Khan (2020) who used an adapted version of Peyton’s 4 stage process (1998) to deliver small group clinical skills teaching online in a UK medical school. The author used peer tutors to teach small groups of medical students skills such as examination skills using an adapted Peyton’s process. Students were then encouraged to practice these skills on themselves and others if safe to do so. Students then received feedback and oversight from the tutors and lecturers in the unit.

In another approach, Wallace (2020) sent students resource packs with clinical skills equipment to their homes and used a flipped learning approach to teach a range of practical skills. Wallace and her team asked students to use, for example, cucumbers for bladder catheterisation and bananas for suturing practice. This novel approach provided students with much needed motor skills practice and highlighted the importance of actually doing skills as opposed to solely watching them or discussing them in an online format. There were however challenges with this approach; Wallace’s team were unable to send packs to students outside of the UK and struggled to find suitable teaching spaces in their own homes to deliver these sessions.

From our experience we suggest using an approach that enables students to reflect on a skill from multiple perspectives. For us this involved using multiple online learning modalities (videos, written resources, live online sessions, discussion forums) and encouraging students to practice part-task examinations (for example pulse rate, heart rate and rhythm and percussion) on themselves and others if safe to do so.

## Tip 6: Find or create video and other media to suit your learners’ needs

Appropriate use of a variety of different media can be an effective way to vary the stimulus for learners and encourage active engagement online. The key is to ensure that your instructional design and pedagogical approach drives your choice of media, and not the other way around ( Goh, 2019). For example, when teaching physical examination using a blended approach, think how a video could be incorporated into an adapted version of Peyton’s 4 step model ( Khan, 2020). Could you show the entire skill before then breaking down into small chunks of video, showing key steps with simultaneous commentary?

There are many excellent open access videos and other media that you can use for teaching online. Sometimes videos don’t demonstrate the exact skill we had in mind for our students, however they can be very useful as material to critique in online discussions. If you have the resources, professionally produced videos of skills, procedures and consultations can be tailor-made for your learners. But it is also possible to create valuable media resources using more accessible technology such as a mobile phone camera, and then using widely available free video-editing software to add voice-overs, illustrations and written guidance. The learning value lies less in the production quality and more in the thought that has gone into the pedagogical intent.

## Tip 7: Use the multi-disciplinary expertise within your department

In order to overcome the difficulties associated with moving curriculum content online it is a good idea to use the varied expertise available to you. For example, our unit has a range of staff from GPs to those with a background in obstetrics, sexual health and psychology. Our varied backgrounds were used to engage students in discussions related to case scenarios, particularly in providing varied perspectives on possible clinical contexts. This has been shown to be beneficial in both promoting patient care and learning (Coventry, Coventry, and Coventry, 2017).

We would also suggest providing varied online content that highlights the varying skill sets with any one team and try to create a safe environment where teachers and learners can explore students’ ideas and concerns. In the example above curiosity was piqued through discussions about simulated cases as students were able to question us about things they may have found challenging i.e. ‘how would you deal with a patient in pain?’.

## Tip 8: Support and learn from each other

The importance of teamwork in the creation and delivery of a sleek, user friendly, online pedagogical tool cannot be overstated. It is vital therefore that educators, administrators and technical staff share good practice to foster and develop online content ( Watson and Fardinpour, 2017). Across education, the appearance and consequences of COVID-19 necessitated a sudden transfer of learning to an online format and with it a sudden need for robust teamworking mechanisms to make this possible.

In our unit, particular challenges included the fact that there was a relative lack of technical expertise with respect to online learning platforms and tools. This was overcome through training and exploration but as we learned we also provided mutual support to each other during the entirety of the module development process. There were also constant adjustments to the module based on feedback as alluded to by Sandars and Lafferty (2010). As a clinical and communication skills unit, by sharing our expertise in technical and pedagogical skills not just within our unit but also outside of it through presentations within our school and individual and group training sessions with other departments, we were able to design and deliver an online course on extremely short notice, benefitting both the students and educators alike.

## Tip 9: Try it out first

We have found that piloting new online sessions is extremely valuable to test out the practicalities of online delivery, from the technology to student dynamics. We rearranged the curriculum to allow us to pilot new sessions with a representative sample of students (we are fortunate to have a second campus outside of the UK with a smaller cohort of students studying the same curriculum, but more flexibility in timing and sequencing of delivery). Students on the pilot sessions benefitted by having a higher tutor-student ratio, as all faculty were involved, and from novel teaching interventions, some of which we realised could not feasibly be rolled out to a larger cohort. These pilots identified a range of previously unrecognised issues, including problems with connectivity and bandwidth, group size and dynamic, and smartphone/handheld tablet compatibilities. Some sessions were further developed and improved after the pilots, and faculty felt more confident in navigating their way around the new online learning space.

## Tip 10: Embrace remote consulting as a new skill set

In early 2020, remote consulting was the future; now, accelerated by the Covid-19 crisis, it’s very much the present and likely to be here to stay. With the online teaching medium mirroring current clinical practice, video consulting in particular has become a key component of the modern communication skills curriculum. Whilst consulting in person and consulting via video share similar skills, video consulting is not simply a face to face consultation with technology bolted on ( King and Smith, 2020) and therefore there are specific skills and approaches students need to learn in preparation for placements in the future. We have worked closely with our actor colleagues and clinicians to develop and deliver video consulting teaching to highlight some of these key skills. For example, awareness of eye contact - looking at the camera or the screen; paying particular attention to non-verbal cues; summarising; and speaking clearly with breaks in flow of speech to avoid talking over each other ( Greenhalgh, 2020; Centre for Telemedicin and Tele Health, 2020). In addition, students need clear orientation to the professionalism and ethical issues that online consulting raises.

## Tip 11: Be prepared to fail at some things

Medical errors are a source of significant worry for medical practitioners ( Fischer *et al*. 2006). Yet we know that medical errors are commonplace ( Vincent, Neale, and Woloshyowych, 2001) and professionals can learn a great deal from discussing their mistakes ( Millwood, 2014). Just as medical practitioners are embracing the value of learning together from their mistakes, medical educators need to adopt a similar openness to failings in teaching and learning.

An experienced group facilitator can find themselves humbled when faced with teaching online. Online teaching draws on IT skills that aren’t routinely needed in the classroom. The Expert to Novice model ( Dunbar, Kawar, and Scruth, 2019) describes the process a person goes through when practicing in a new domain, such as teaching online. The model suggests we should embrace our status as novices and be open to learning from our mistakes. COVID-19 and those online learning experiences that preceded the pandemic has forced medical educators to reassess their relationship with curriculum content and its delivery. This can be a challenging process and one that, as described in the tip above, requires honest self-reflection and peer review.

To realise this aim we suggest strategies that focus on developing pedagogy and team work so as to enable educators to continue to develop their teaching approach in a supportive environment despite failures and errors that occur. Strategies such as keeping reflective notes about sessions, reviewing student feedback in real time and organising regular team debriefs have been useful in our department over this challenging period. This approach has also cemented many working relationships between colleagues, which in turn has positively impacted on other module development processes.

## Tip 12: Collect data and evaluate outcomes

New technologies offer unique opportunities for promoting reflective learning and evaluating the impact of educational interventions. Many authors argue a prerequisite for capitalising on these tools requires creating a ‘reflective practicum’ by which academics are moulded into reflective practitioners ( Maor, 2003; Laurillard, 2002). Reflection requires an active effort to understand and evaluate the process of teaching and learning as well as opening up opportunities to learn about oneself. ( Bonk *et al*., 2001) recommends online educators to engage in four areas; pedagogy, social interaction, management and technology. Embedding and participating in multiple feedback mechanisms can therefore help gather new insights and perspectives.

A helpful tip for both individual and group reflection is keeping a transparent audit trail of lessons learnt and creating a structured method for gathering feedback from participants throughout the course. This can be done through a feedback session at the end of a teaching session and university-wide student surveys. Providing opportunities to discuss thoughts and feedback supports educators in further modelling reflective practice and provides an avenue for restructuring programs. A road map can also be created to show how and when various goals were achieved. This is a useful mechanism to gain team members buy-in before sharing more broadly with other stakeholders. The process also provides an opportunity to celebrate accomplishments and identify items that can be the focus for improvement in subsequent teaching design. This becomes much easier if the entire team feels ownership of the program design and implementation ( Edwards *et al*., 2019).

## Conclusion

Practical subjects such as clinical and communication skills are difficult to teach effectively in online. However, in this paper the authors outline twelve tips for doing so highlighting the need for a robust pedagogical foundation and use of the extensive interactive opportunities that online learning can provide educators with. We consider the importance of the educational multi-disciplinary team in providing holistic support and expertise for learning, evaluation and reflection from both students and staff, and strategies for managing failure in the online environment.

## Take Home Messages

• Teaching practical skills such as clinical and communication skills online can be challenging.
• Online learning is an established method of teaching in medical education.
• The online learning environment can be an effective method of teaching clinical and communication skills especially in situations such as the current pandemic where all learning is remote.
• For effective clinical and communication skills teaching online educators need to use robust pedagogical foundations to frame teaching, which is student-focussed, utilises institution-supported elearning technologies, supports the educational MDT, evaluates and reflects on educational resources, and learns from failure.

## Notes On Contributors

**Helen L Bintley** MBChB DFSRH LoCSDI LoCIUT MA SFHEA, is a Senior Lecturer, Head of Quality in Teaching and Learning and Joint Lead for Clinical Skills (London) at Barts and The London School of Medicine and Dentistry, Queen Mary University of London.

**Graham Easton**, MB BS, MEd, MSc, FRCGP, is a General Practitioner and Professor of Clinical Communication Skills at Barts and The London School of Medicine and Dentistry, Queen Mary University of London. He is Head of the Clinical and Communication Skills Unit and was formerly a Programme Director for GP Specialty Training at Imperial College School of Medicine.

**Riya** **E. George**, BSc, MSc, MSc, PhD, PGCAP, CPsychol, SFHEA. Lecturer in Clinical Communication Skills and Co-Chair of the Equality, Diversity and Inclusion Committee for the School of Medicine and Dentistry at the Institute of Health Sciences Education, Queen Mary University of London. Riya is on the Board of Directors for the Association of the Study of Medical Education (ASME), a National Committee Member of Diversity in Medicine in Healthcare (DIMAH) and a Committee Member of the General Medical Council’s Equality, Diversity and Inclusion Steering Group.

**Devina Raval** MBBS, BSc, MRCGP, MA, FHEA is a General Practitioner and Senior Clinical Lecturer at Barts and The London, School of Medicine and Dentistry, Queen Mary’s University of London. She currently co-leads Clinical Skills for the MBBS London curriculum at Barts and The London, School of Medicine and Dentistry, Queen Mary’s University of London.

**Harvey Wells** MSc MA SFHEA is a Senior Lecturer in Clinical Communication at Barts and the London School of Medicine, Queen Mary University of London. By background he is a psychotherapist and has specialised in working with patients with comorbid mental health and substance use issues.

**Nisha Ehamparanathan** MBBS, BMedSci, MRCGP, MA, FHEA, is a GP and Lecturer at Barts and the London, Queen Mary University of London. She specialises in the delivery of Clinical skills and has a keen interest in the development of teaching resources.

**Harriet Le Voir** MBBS, DipGum, DFSRH, MRCP, PGcert is a Sexual Health and HIV registrar at the Royal London Hospital. She has worked as a Clinical Skills Teaching Fellow at Barts and the London School of Medicine and Dentistry and continues to contribute as an Honorary Lecturer with medical student education.

**S. Elizabeth Wright** MD CCFP FCFP did her medical training in Canada. After 25 years of Family Practice, she relocated to the UK. She has lectured in the Clinical Communication Skills Unit at Barts and The London School of Medicine and Dentistry, Queen Mary University of London. She is currently an Honorary Lecturer in the Centre for Medical Education within the Institute of Health Sciences Education, Queen Mary University of London.

**Dason Evans** is a Senior Lecturer in Medical Education, Head of Clinical Skills at Barts and the London (currently seconded to the Malta campus), and a part time Sexual Health Doctor. He has a Masters in Health Professions Education and has worked in medical education for the last 20 years. He has particular interests in the teaching, learning and assessment of clinical skills; supporting students in academic difficulty; and helping the development of medical teachers.

**Angela Rowlands** MSc, is a Senior Lecturer at Barts and the London School of Medicine and Dentistry Queen Mary University of London. She is currently Head of Academic and Pastoral Support and Head of the Communications Skills Curriculum at their Malta campus.

**Ashia Shafi** is a clinical lecturer in clinical and communication skills at Barts and the London School of Medicine and Dentistry Queen Mary University of London. Ashia is also a health assessment doctor in London UK and has worked in public health, international development spaces, where she has held senior roles in various countries across South-East Asia and Sub- Saharan Africa.
