# Supplementary material for: Twelve Tips for teaching clinical and communication skills online
Source: MedEdPublish (2016). 2021 Jan 29;10:27. [Version 1] doi: 10.15694/mep.2021.000027.1 (PMC10939600; doi:10.15694/mep.2021.000027.1)
Supplement: Peer review history

# Reviewer report: Reviewer response for version 1

**DOI:** 10.21956/mep.18897.r26994

**Published:** 2021-05-05

**Authors:** Ken Masters

**Affiliations:** 1 Sultan Qaboos University

This review has been migrated. The reviewer awarded 3 stars out of 5 A useful 12-tips paper for teaching clinical and communication skills online. The authors correctly identify the problems of teaching these skills (not easy in a face-to-face situation) in an online environment in response to the demands brought on by COVID-19.The paper is a lovely blend of reference to the literature and theory and practical tips based on personal experience for the many educators who are having to find their way through this ocean of uncertainty. Sometimes, some of the information is broader than is warranted from the title, but it does serve as useful contextual information, and it is frequently difficult to separate the information into small boxes.There are some places in the text, though, when I was looking for a little more detail, and asking “How?”, as, sometimes, even the practical advice was at one level higher than it should be. So, I would find myself saying: “Mmm, nice idea - how exactly would I go about doing that?” While I realise that much might depend upon specific circumstances, I think a lower level of detail would help in places, especially for novices, for whom 12-tips papers are particularly useful.I will leave the specific places up to the authors, but I would look forward to Version 2 of the paper, in which they identified areas where the practical advice could be given some more “how” advice.

Reviewer Expertise:

NA

No decision status is available

# Reviewer report: Reviewer response for version 1

**DOI:** 10.21956/mep.18897.r26993

**Published:** 2021-02-09

**Authors:** Anne Stephenson

**Affiliations:** 1 King's College London

This review has been migrated. The reviewer awarded 4 stars out of 5 This is a very helpful article at this time of Covid-19 when as educators we have all been required to develop our online teaching skills rapidly. Learning clinical skills and communication skills are particularly challenging online. The pedagogical basis for the educational approaches and practical examples are well referenced and the article is well written and easy and quick to read (we all need that just now). Thank you Queen Mary.

Reviewer Expertise:

NA

No decision status is available

# Reviewer report: Reviewer response for version 1

**DOI:** 10.21956/mep.18897.r26996

**Published:** 2021-02-01

**Authors:** Samar Ahmed

**Affiliations:** 1 Ain Shams University Faculty of Medicine

This review has been migrated. The reviewer awarded 3 stars out of 5 Thank you for the elaborate work. The aim of this work is clear. The title is informative and relevant.The references are relevant, recent, referenced correctly and appropriate key studies are included. The research are is clear and well discussed with a very good narration of what is already known about the topic. Tables and figures relevant and clearly presented.The results are impactful given the importance of the tips offered that are basically a mixture of pedagogy and tools. The article is consistent with itself and offers a good array of tips that can be used by developers, course planners and teachers at the same time. Points to consider:There is no reference in the introduction section to how the authors reached this consensus twelve tip document apart from the sentence: "The following tips have been assembled by the members of one teaching team in a clinical and communication skills unit in a UK medical school. They draw on their extensive experience of using technology-enhanced learning to offer advice for use in and beyond the current situation". The author might consider giving more insight on the methodology used to assemble information and reach the common understanding between authors. Please consider adding this study to the existing body of references in your work. https://doi.org/10.1016/j.hpe.2017.10.003The conclusions drawn in this work are a result of opinions that are expert opinions and that are important yet need a little more referencing or methodological reasoning in some places eg. tip 9 z

Reviewer Expertise:

NA

No decision status is available

# Reviewer report: Reviewer response for version 1

**DOI:** 10.21956/mep.18897.r26995

**Published:** 2021-01-31

**Authors:** Katherine Joekes

This review has been migrated. The reviewer awarded 4 stars out of 5 The engaging article addresses a challenge faced by all undergraduate medical education programmes during the Covid-19 pandemic. It describes how one institution approached this challenge, highlighting the importance of collaboration, flexibility and innovation. In addition, it recognises that new educational practices, which embrace more online learning, might be sustained in the future. This gives the paper relevance for the years ahead. The authors have chosen to take a blended approach to the formulation of the Twelve Tips by focusing on both pedagogical principles and common-sense application, which at times forces the reader to shift gears from the ‘bigger picture’ to the more mundane. The core question of whether students are able to develop appropriate and safe levels of competencies on the basis of online teaching, lies beyond the scope of this paper and remains to be answered!

Reviewer Expertise:

NA

No decision status is available
